# Supplementary material for: Nutritional sex-specificity on bacterial metabolites during mosquito (Aedes aegypti) development leads to adult sex-ratio distortion
Source: Commun Biol. 2024 Dec 2;7:1603. doi: 10.1038/s42003-024-07319-7 (PMC11612200; doi:10.1038/s42003-024-07319-7)
Supplement: Supplementary file 3 — Description of Additional Supplementary Files [file 42003_2024_7319_MOESM3_ESM.pdf]

Description of Additional Supplementary Files

File name: Supplementary Data 1

Description: Raw data for all figures.

File Name: Supplementary Data 2

Description: Detailed results of statistical tests.
