# Supplementary material for: Nutritional sex-specificity on bacterial metabolites during mosquito (Aedes aegypti) development leads to adult sex-ratio distortion
Source: Commun Biol. 2024 Dec 2;7:1603. doi: 10.1038/s42003-024-07319-7 (PMC11612200; doi:10.1038/s42003-024-07319-7)
Supplement: Supplementary file 6 — Reporting summary [file 42003_2024_7319_MOESM6_ESM.pdf]

nature portfolio

Corresponding author(s): Mathilde Gendrin

Last updated by author(s): Nov 8, 2024

Reporting Summary

Nature Portfolio wishes to improve the reproducibility of the work that we publish. This form provides structure for consistency and transparency in reporting. For further information on Nature Portfolio policies, see our Editorial Policies and the Editorial Policy Checklist.

Statistics

For all statistical analyses, confirm that the following items are present in the figure legend, table legend, main text, or Methods section.

n/a	Confirmed	
☐	☒	The exact sample size (*n*) for each experimental group/condition, given as a discrete number and unit of measurement
☐	☒	A statement on whether measurements were taken from distinct samples or whether the same sample was measured repeatedly
☐	☒	The statistical test(s) used AND whether they are one- or two-sided<br>*Only common tests should be described solely by name; describe more complex techniques in the Methods section.*
☒	☐	A description of all covariates tested
☐	☒	A description of any assumptions or corrections, such as tests of normality and adjustment for multiple comparisons
☐	☒	A full description of the statistical parameters including central tendency (e.g. means) or other basic estimates (e.g. regression coefficient) AND variation (e.g. standard deviation) or associated estimates of uncertainty (e.g. confidence intervals)
☐	☒	For null hypothesis testing, the test statistic (e.g. *F*, *t*, *r*) with confidence intervals, effect sizes, degrees of freedom and *P* value noted<br>*Give P values as exact values whenever suitable.*
☒	☐	For Bayesian analysis, information on the choice of priors and Markov chain Monte Carlo settings
☒	☐	For hierarchical and complex designs, identification of the appropriate level for tests and full reporting of outcomes
☒	☐	Estimates of effect sizes (e.g. Cohen's *d*, Pearson's *r*), indicating how they were calculated

Our web collection on statistics for biologists contains articles on many of the points above.

Software and code

Policy information about availability of computer code

Data collection	No software was used for data collection.
Data analysis	GraphPad Prism (version 10.0.2); R (version 4.3.0); FastQC (v 0.11.9); Trimmomatic (v 0.40); HISAT2 (v 2.2.1); featureCounts (v 1.6.4); DESeq2 (v 1.40.1)

For manuscripts utilizing custom algorithms or software that are central to the research but not yet described in published literature, software must be made available to editors and reviewers. We strongly encourage code deposition in a community repository (e.g. GitHub). See the Nature Portfolio guidelines for submitting code & software for further information.

Data

Policy information about availability of data

All manuscripts must include a data availability statement. This statement should provide the following information, where applicable:

- Accession codes, unique identifiers, or web links for publicly available datasets
- A description of any restrictions on data availability
- For clinical datasets or third party data, please ensure that the statement adheres to our policy

The raw RNA sequencing data underlying Figure 2 and Supplemental Figure S2 and S3 are available in the Sequencing Read Archive (SRA) under the Bioproject ID PRJNA1155297. The R code to run statistical test is available at https://zenodo.org/doi/10.5281/zenodo.13742138. Source data are provided in Supplementary Data 1.

Research involving human participants, their data, or biological material

Policy information about studies with human participants or human data. See also policy information about sex, gender (identity/presentation), and sexual orientation and race, ethnicity and racism.

Reporting on sex and gender	N/A
Reporting on race, ethnicity, or other socially relevant groupings	N/A
Population characteristics	N/A
Recruitment	N/A
Ethics oversight	N/A

Note that full information on the approval of the study protocol must also be provided in the manuscript.

Field-specific reporting

Please select the one below that is the best fit for your research. If you are not sure, read the appropriate sections before making your selection.

☒ Life sciences ☐ Behavioural & social sciences ☐ Ecological, evolutionary & environmental sciences

For a reference copy of the document with all sections, see nature.com/documents/nr-reporting-summary-flat.pdf

Life sciences study design

All studies must disclose on these points even when the disclosure is negative.

Sample size

Sample sizes were chosen based on previously published literature for the performed assays:
- larval development: (1); (2); (3)
- CFU measurements: (2); (3)
- transcriptome sequencing: (2); (3). We aimed for 60 individuals because MG's previous work on Drosophila larvae (4) based on 30 individuals/samples was not precise enough to recover a perfect clusterisation of samples by replicate while other studies based on 60 Drosophila or mosquito guts/sample, did (3, 5). Although we aimed at 60 larvae per condition/replicate and more larvae were collected (~150 per condition/replicate), some samples were excluded from the sequencing because of impossibility to determine genetically larval sex.

(1) Coon, K. L., Vogel, K. J., Brown, M. R. & Strand, M. R. Mosquitoes rely on their gut microbiota for development. Mol. Ecol. 23, 2727–2739 (2014).
(2) Vogel, K. J., Valzania, L., Coon, K. L., Brown, M. R. & Strand, M. R. Transcriptome sequencing reveals large-scale changes in axenic Aedes aegypti larvae. PLoS Negl. Trop. Dis. 11, e0005273 (2017).
(3) Romoli, O., Schönbeck, J.C., Hapfelmeier, S. et al. Production of germ-free mosquitoes via transient colonisation allows stage-specific investigation of host–microbiota interactions. Nat Commun 12, 942 (2021).
(4) Gendrin, M., Zaidman-Rémy, A., Broderick, N.A., Paredes, J., Poidevin, M., Roussel, A., Lemaitre, B. Functional Analysis of PGRP-LA in Drosophila Immunity. PLoS ONE 8(7): e69742 (2013).
(5) Buchon N, Broderick NA, Poidevin M, Pradervand S, Lemaitre B. Drosophila intestinal response to bacterial infection: activation of host defense and stem cell proliferation. Cell Host Microbe. Feb 19;5(2):200-11. doi: 10.1016/j.chom.2009.01.003. PMID: 19218090 (2009).

Data exclusions

Some RNA larval sample were excluded from the sequencing because of impossibility to determine genetically their sex. Moreover, transcriptome samples from the third replicate were excluded because the post-sequencing quality control spotted higher male RNA counts in samples marked as females, making the analysis impossible. Additionally, the data of the vitamin x1 condition was excluded because of low alignment rates. This information is included in the supplementary material section.

Replication

At least 3 replicates on different egg-batches were performed for all experiments. All results were successfully replicated.

Randomization

Larvae from the same egg batch were randomly allocated into the experimental groups.

Blinding

Investigators were not blinded because quantification involved either automatic analysis (transcriptomic analysis, fluorescence identification of male larvae) or was not subject to investigator bias (CFU counts, development). Moreover, the experimental setup did not allowed to complete blind operators (i.e. vitamin solutions have different colors depending on the concentration and the type of vitamin) and we considered that the increase in experimental complexity linked to blinding was not useful.

Reporting for specific materials, systems and methods

We require information from authors about some types of materials, experimental systems and methods used in many studies. Here, indicate whether each material, system or method listed is relevant to your study. If you are not sure if a list item applies to your research, read the appropriate section before selecting a response.

Materials & experimental systems

n/a	Involved in the study	
☒	☐	Antibodies
☒	☐	Eukaryotic cell lines
☒	☐	Palaeontology and archaeology
☐	☒	Animals and other organisms
☒	☐	Clinical data
☒	☐	Dual use research of concern
☒	☐	Plants

Methods

n/a	Involved in the study	
☒	☐	ChIP-seq
☒	☐	Flow cytometry
☒	☐	MRI-based neuroimaging

Animals and other research organisms

Policy information about studies involving animals; ARRIVE guidelines recommended for reporting animal research, and Sex and Gender in Research

Laboratory animals	Aedes aegypti New Orleans strain and Aaeg-M GSS strain
Wild animals	The study did not involve wild animals
Reporting on sex	Analyses were performed on both mosquito sexes and the sex is always specified when possible.
Field-collected samples	N/A
Ethics oversight	No protected animal was used for the experiments described in this paper. For the maintenance of the mosquito colony, mosquitoes were blood-fed on mice. Protocols have been validated by the French Direction générale de la recherche et de l'innovation, ethical board # 089, under the agreement # 973021.

Note that full information on the approval of the study protocol must also be provided in the manuscript.

Plants

Seed stocks	N/A
Novel plant genotypes	N/A
Authentication	N/A
